# A life-threatening complication of hydatid cyst: tension pneumothorax

**DOI:** 10.1590/0037-8682-0116-2023

**Published:** 2024-05-06

**Authors:** Yener Aydin, Mesut Ozgokce, Fatma Durmaz

**Affiliations:** 1 Ataturk University, Medical Faculty, Department of Thoracic Surgery, Erzurum, Turkey.; 2 Van Yuzuncu Yil University, Medical Faculty, Department of Radiology, Van, Turkey.

**FIGURE 1:**
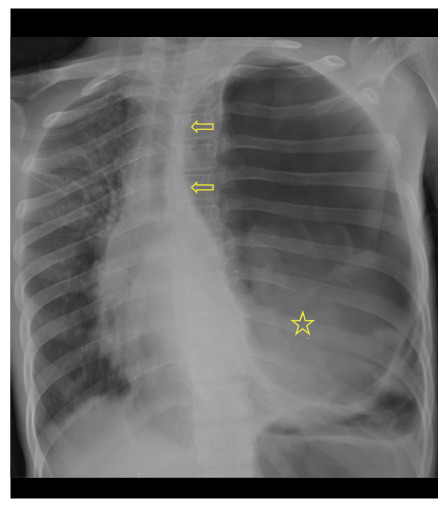
Chest radiograph showing a tension pneumothorax on the left, mediastinal shift to the right (arrows), and a ruptured hydatid cyst (asterisk).

A 12-year-old boy was brought to the emergency department with chest pain, difficulty in breathing, rapid heart rate, cyanosis, and fainting. Chest radiography revealed a tension pneumothorax (Figure 1). Subsequently, a chest drain was inserted to relieve this into the left lung, followed by an emergency surgery. The surgeons performed left thoracotomy, cystotomy, and capitonnage of a large ruptured hydatid cyst in the upper lobe of the left lung. The patient was discharged without complications on the post-operative tenth day.

Hydatid cyst disease is a benign clinical condition that is easy to diagnose and treat in endemic areas^1^. However, pulmonary hydatid cyst rupture can lead to life-threatening complications^2^. Unlike other causes, tension pneumothorax due to a pulmonary hydatid cyst requires surgical and antiparasitic treatment to prevent recurrence, unlike other causes^3^. In such cases, tension pneumothorax should be controlled, and hydatid cysts should be treated surgically as soon as possible. Pulmonary hydatid cysts should be considered as a rare cause of tension pneumothorax.
